# Aging Leads to Elevation of O-GlcNAcylation and Disruption of Mitochondrial Homeostasis in Retina

**DOI:** 10.1155/2014/425705

**Published:** 2014-05-29

**Authors:** Lin Zhao, Zhihui Feng, Xuan Zou, Ke Cao, Jie Xu, Jiankang Liu

**Affiliations:** ^1^Center for Mitochondrial Biology and Medicine, The Key Laboratory of Biomedical Information Engineering of the Ministry of Education, School of Life Science and Technology and Frontier Institute of Science and Technology, Xi'an Jiaotong University, Xi'an 710049, China; ^2^Center for Translational Medicine, The Key Laboratory of Biomedical Information Engineering of the Ministry of Education, School of Life Science and Technology and Frontier Institute of Science and Technology, Xi'an Jiaotong University, Xi'an 710049, China

## Abstract

Retina is particularly susceptible to aging as oxidative damage accumulates within retina, leading to age-related retinal dysfunction or even visual loss. However, the underlying mechanisms still remain obscure and effective therapeutic strategy is urgently in need. Here, we quested for the answer particularly focusing on mitochondrial homeostasis and O-GlcNAcylation in rat retina. By comparing expression of electron transfer chain complexes and key factors in mitochondrial biogenesis and dynamics in retinas of aged and young Sprague-Dawley rats, we found that mitochondrial Complex I, II, IV and V were increased in aged retina with decreased mtTFA and Mfn2. Also, we noticed that p38 and JNK of MAPK signaling were substantially more activated in aged retina, suggesting stress induction. In addition, we found that pan-O-GlcNAcylation was remarkably stronger with lower OGA expression in aged retina. To further elucidate the roles of Mfn2 and O-GlcNAcylation, we employed ARPE-19 cells and found that ATP production, oxygen consumption, and mitochondrial membrane potential were reduced and ROS level was increased by Mfn2 knockdown, while treating with PUGNAc or UDP-GlcNAc heightened oxygen consumption and reduced ROS. Our results suggest disrupted mitochondrial homeostasis may increase oxidative stress; yet enhanced O-GlcNAcylation might defend against oxidative stress and promote mitochondrial respiration in aged retina.

## 1. Introduction

The retina is particularly susceptible to aging, since vital components within retina such as retinal pigment epithelium (RPE) and photoreceptors are highly metabolically active and are nondividing cells; thus, oxidative damage derived from high oxygenation and high dose of exposure to short wavelength visible light is cumulative, resulting in aging-associated retinal dysfunction, visual decline, or even visual loss, such as age-related macular degeneration (AMD), the leading cause of blindness among the elderly [1, 2]. A wide spectrum of investigations attempting to reveal the mystery of retinal aging has been carried out, ranging from the changes of the ultrastructure [3] to early cellular or molecular events; yet efficacious remedy to related retinal diseases is still lacking due to a far incomplete understanding of the underlying mechanisms.

It has been long acknowledged that, on the central stage of aging and the wide spectrum of age-related degenerative diseases, there stands one of the major contributors, mitochondrial dysfunction. It includes increased disorganization of mitochondrial structure, decline in mitochondrial energy metabolism, enhanced mitochondrial oxidative stress, and the resulting damage, especially the accumulation of mtDNA damage [4]. A growing body of evidence has confirmed the association between mitochondrial dysfunction and age-related retinal pathophysiology [5]; nevertheless, most of them mainly focused on mitochondrial genomic instability [6–11] and mitochondrial dysfunction originating from oxidative stress [11–13]. However, besides the well-documented age-related compromise in retinal mtDNA integrity, little is known so far regarding changes of mitochondrial biogenesis or dynamics which occurred during retinal aging. There has been only limited evidence hinting at the decline of mitochondrial content focalized in the context of AMD. Feher et al. performed electron microscopic and morphometric studies on human RPE from AMD and from age- and sex-matched controls and saw significant decrease in number and area of mitochondria as well as loss of cristae and matrix density with increasing age, and greater decreases were found in AMD than in normal aging [14]. Ferrington group analyzed the subproteome of mitochondria isolated from RPE of human donor eye with different progressive stages of AMD by two-dimensional gel electrophoresis and succeeding mass spectrometry and found that decreased content of *α*-, *β*-, and *δ*-ATP synthase subunits, altered mitochondrial translation and import of nuclear-encoded proteins is involved in etiology of AMD [15].

Although in the light of the above sparing evidence a drop in mitochondrial number or content during human retinal aging especially in case of AMD might be implied, it requires further study to elaborate the change of mitochondria in retinal aging and to explore the underlying molecular mechanisms. In our previous study reported recently, we showed that lipid accumulates with aging in muscle, heart, and liver of rats, which might be attributed to downregulated peroxisome proliferator-activated receptor gamma coactivator 1-*α* (PGC1-*α*), a key factor in mitochondrial biogenesis, disrupted mitochondrial dynamics owing to decreased expression of Mfn2, and suppressed autophagy [16]. We have also obtained retinas from those 5-month-old and 25-month-old rats and compared expression profile of mitochondrial electron transport chain (ETC) complexes subunits as well as key molecules involved in mitochondrial biogenesis and dynamics.

O-GlcNAcylation is a posttranslational modification with rising recognition of its role in sensing and responding to fluctuant environmental nutrient and stress conditions by regulating fundamental protein functions encompassing protein interaction, oligomerization, stability, localization, and enzymatic activity during the past three decades [17, 18]. However, little is known on whether O-GlcNAcylation has any part in retinal aging and related diseases, except a recent study suggesting that O-GlcNAcylation was involved in the progression of diabetic retinopathy [18]. Therefore, in the current study, we would also like to investigate the alternations of O-GlcNAcylation during retinal aging and explore the role of O-GlcNAc cycling in association with mitochondrial regulation in retinal aging.

## 2. Materials and Methods

### 2.1. Chemicals and Reagents

Antibodies to PGC-1*α*, mtTFA, Mfn-1, Mfn-2, Drp-1, OPA-1, p-Erk1/2, Erk1/2, p-JNK, JNK, p-p38, p38, OGA, OGT, and O-GlcNAc were from Santa Cruz Biotechnology (Santa Cruz, CA); anti-GAPDH was from Cell Signaling Technology (Danvers, MA); and antibodies to Complexes I (NDUFS3), II (subunit 30 kDa), III (subunit core 2), IV (subunit I), and V (subunit alpha) were from Invitrogen (Carlsbad, CA). Other chemicals and reagents were purchased from Sigma if not otherwise indicated.

### 2.2. Animals

Sprague-Dawley (SD) male rats were purchased from a commercial breeder (SLAC, Shanghai). The rats were housed in a temperature (24–26°C) and humidity (60%) controlled animal room and maintained on a 12 h light/12 h dark cycle (light on from 08:00 a.m. to 08:00 p.m.) with free access to food and water throughout the experiments. Four-week-old male rats weighing 180–200 g were used to start the experiments. After reaching 25 months and 5 months of age (old and young group, resp.), the animals were sacrificed and retina samples were collected for analysis. All of the procedures were performed in accordance with the United States Public Health Services Guide for the Care and Use of Laboratory Animals, and all efforts were made to minimize the suffering and the number of animals used in this study.

### 2.3. Cell Culture

A human ARPE-19 cell line was purchased from ATCC and was cultured in DMEM-F12 medium supplemented with 10% fetal bovine serum, 0.348% sodium bicarbonate, 2 mM L-glutamine, 100 U/mL penicillin, and 100 *μ*g/mL streptomycin. Cell cultures were maintained at 37°C in a humidified atmosphere of 95% air and 5% CO_2_. Medium was changed every two days. ARPE-19 cells were used within 10 generations.

### 2.4. siRNA Transfection

siRNA transfection was performed using the target sequence 5′-3′ for human Drp1, Mfn1, and Mfn2 siRNA. ARPE-19 cells were seeded at 1.5 × 10^5^ cells per well in six-well plates. The transfection, using Lipofectamine 2000, was performed as described in the supplier's manual. Briefly, appropriate amounts of siRNA and 5 *μ*L Lipofectamine 2000 in 250 *μ*L serum-free DMEM/12 medium were prepared in separate RNase-free tubes. After 5 minutes of incubation, the siRNA and Lipofectamine were mixed and incubated for another 20 minutes and then added to each well. After incubation with 100 pmol siRNA per well for 48 h, cells were collected for further analysis.

### 2.5. Intracellular Adenosine 5′-Triphosphate (ATP) Level Measurement

Cells were cultured in 6-well plates. After treatment, cells were lysed by 0.5% Triton X-100, in 100 mM glycine buffer, pH 7.4. Intracellular ATP level assays were carried out with an ATP bioluminescent assay kit (Sigma). ATP is consumed and light is emitted when firefly luciferase catalyzes the oxidation of D-luciferin.

### 2.6. Assay for Oxygen Consumption Capacity

Oxygen consumption capacity was determined with the BD Oxygen Biosensor System (BD Biosciences). Plates were sealed and scanned by a fluorescence spectrometer (Fluoroskan Ascent, Thermo Fisher Scientific Inc., Waltham, MA) at 1-minute intervals for 60 minutes at an excitation wavelength of 485 nm and emission wavelength of 630 nm.

### 2.7. JC-1 Assay for Mitochondrial Membrane Potential (MMP)

ARPE-19 cells were cultured in 24-well plates. After Mfn2 siRNA transfection for 48 h, MMP was detected with JC-1. For quantitative fluorescence measurement, cells were incubated with JC-1 staining and scanned using a microplate fluorometer (Fluoroskan Ascent, Thermo Fisher Scientific Inc., Waltham, MA) at 488 nm excitation and 535 nm and 590 nm emissions wavelengths to measure green and red JC-1 fluorescence, respectively. The red/green fluorescence intensity ratio reflects mitochondrial membrane potential.

### 2.8. Determination of Reactive Oxygen Species (ROS) Generation

The generation of intracellular ROS was determined by fluorescence of 2′,7′-dichlorofluorescein (DCF-DA), upon oxidation of nonfluorescent, reduced, DCFH. The fluorescence intensity of the supernatant was measured with a microplate fluorometer (Fluoroskan Ascent, Thermo Fisher Scientific Inc., Waltham, MA) at 488 nm excitation and 535 nm emission. Cellular oxidant levels were expressed as relative DCF fluorescence per *μ*g of protein (BCA method).

### 2.9. Western Blot Analyses

Retinal tissues were rinsed with PBS, trimmed off attached connective tissues, cut into pieces with scissors prior lysis, and then homogenized in Western and IP lysis buffer (Beyotime, China). The homogenates were centrifuged at 13,000 g for 15 min at 4°C. The supernatants were collected and protein concentrations were determined with the BCA Protein Assay kit (Pierce 23225). Equal aliquots (20 *μ*g) of protein samples were applied to 10% SDS-PAGE gels, transferred to pure nitrocellulose membranes (PerkinElmer Life Sciences, Boston, MA, USA), and blocked with 5% nonfat milk. The membranes were incubated with antibodies at 4°C overnight. Then the membranes were incubated with anti-rabbit or anti-mouse antibodies at room temperature for 1 hour. Chemiluminescent detection was performed by an ECL western blotting detection kit (Pierce). The results were analyzed by Quantity One software to obtain the optical density ratio of target protein to beta-Actin.

### 2.10. Statistical Analysis

All data are reported as the means ± S.E.M. The statistical analysis was performed using *t*-test analysis or one-way ANOVA followed by LSD* post hoc* analysis. In all comparisons, the level of significance was set at *P* < 0.05.

## 3. Results

### 3.1. Expression of Mitochondrial Complexes Increases While Expression of Mitochondrial Biogenesis Regulators Decreases in Aged Retinas Compared with Young Ones

**Figure 1:**
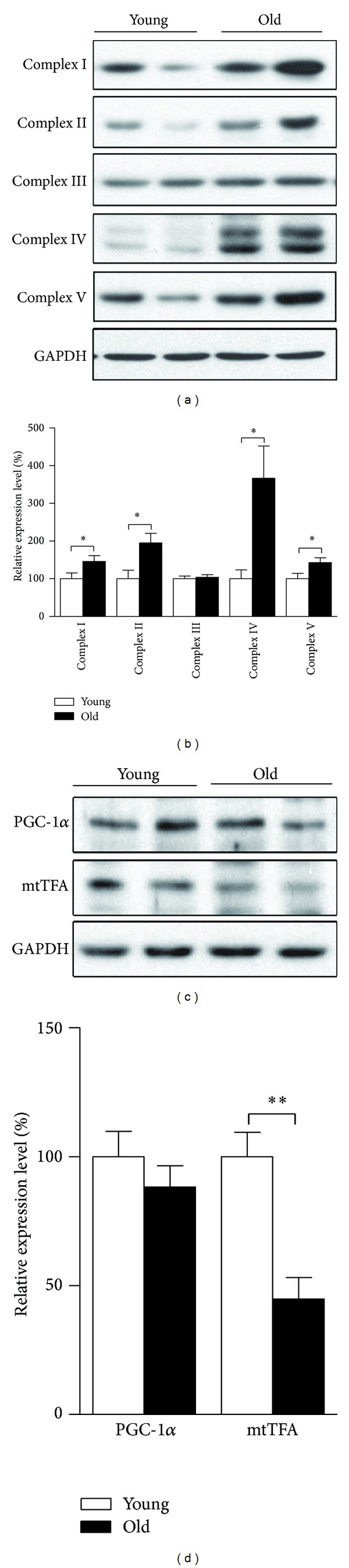
Mitochondrial biogenesis status in retinas of young and old rats. SD rats aged five months (young) and 25 months (old) were sacrificed; total proteins of retina tissues were prepared for analysis. Mitochondrial complexes expressions including Complexes I, II, III, IV, and V were detected by Western blot ((a) Western blot image and (b) statistical analysis). Mitochondrial biogenesis regulators, PGC-1*α* and mtTFA, were analyzed by Western blot ((c) Western blot image and (d) statistical analysis). The values are the means ± S.E.M from 8 rats per group. *P < 0.05 and **P < 0.01 as calculated by Student's *t*-test.

To elucidate the changes of mitochondria which occurred in rat retina during aging, retinas were isolated and whole protein extracts of retinas were analyzed. Protein expression levels of ETC complexes, the core components of mitochondria, were analyzed and Complexes I, II, IV, and V were all found significantly higher in aged retinas (Figures 1(a) and 1(b)). The expression of the crucial mitochondrial biogenesis regulator PGC-1*α* was not different between young and old rats (Figures 1(c) and 1(d)), while the other regulator, mitochondrial transcription factor A (mtTFA), the key activator of mitochondrial transcription and essential player in mitochondrial genome replication [19], was found downregulated considerably in aged retina (Figures 1(c) and 1(d)), suggesting aging-associated decline in retinal mitochondrial biogenesis.

### 3.2. Changes in Mitochondrial Dynamic Regulators during Retinal Aging

**Figure 2:**
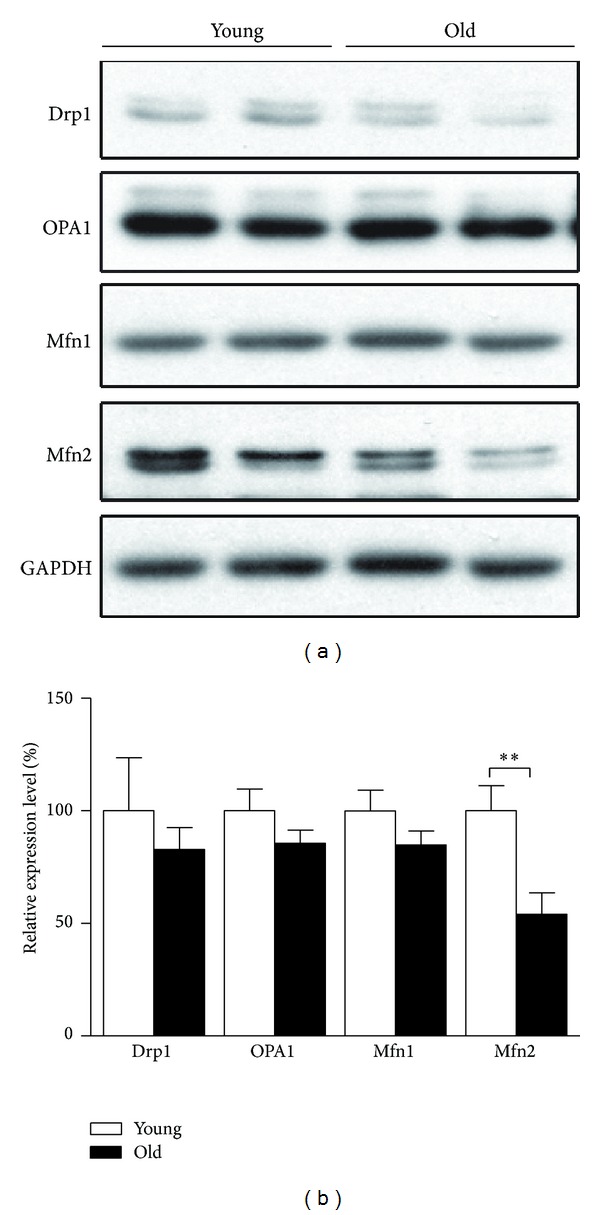
Changes of mitochondrial dynamic regulators in retinas of young and old rats. SD rats aged five months (young) and 25 months (old) were sacrificed; total proteins of retina tissues were prepared for analysis. Expressions of mitochondrial dynamic regulators including Drp1, OPA1, Mfn1, and Mfn2 were analyzed by Western blot ((a) Western blot image and (b) statistical analysis). The values are the means ± S.E.M from 8 rats per group. *P < 0.05 and **P < 0.01 as calculated by Student's *t*-test.

The increased mitochondrial complexes but declined mitochondria biogenesis directed our attention to investigate whether mitochondrial degradation is impaired. Orderly disposal of defective mitochondria requires well-functioning mitochondrial dynamics, a tightly regulated homeostasis between mitochondrial fusion and fission. We compared the expression levels of core proteins of the conserved molecular machinery mediating mitochondrial fusion and fission between young retina tissues and aged ones, including Opa1 in the intermembrane space, Mfn1 and Mfn2 in the outer membrane, key players engaged in mitochondrial fusion, and dynamin-related protein 1 (Drp1), which are recruited from cytosolic pool to mitochondrial surfaces and assembled into potential scission sites together with Fis1 [20, 21]. Among those, Mfn2, a mitofusin protein mediating fusion through their active GTPase domain by tethering opposing mitochondrial membranes together [21], was found remarkably downregulated in aged retina, though no significant changes were detected on all other three molecules that have been examined, Drp1, OPA1, and Mfn1 (Figures 2(a) and 2(b)). This should contribute to disrupted mitochondrial fusion and the resulting compromised mitochondrial integrity and quality.

### 3.3. Mfn2 Knockdown Impairs Mitochondrial Function and Induces Oxidative Stress in ARPE Cells

**Figure 3:**
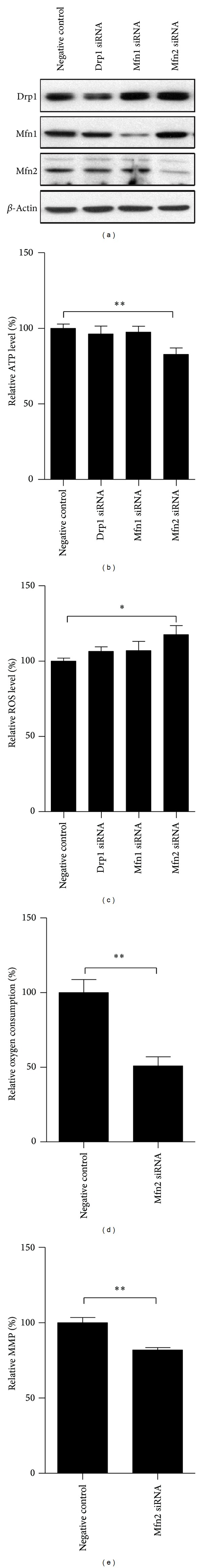
Effects of Drp1, Mfn1, or Mfn2 knockdown on mitochondrial function in ARPE-19 cells. ARPE-19 cells were transfected with Drp1, Mfn1, or Mfn2 siRNA, respectively for 48 h; protein expressions were confirmed by Western blot (a), cellular ATP content (b), ROS content (c), and oxygen consumption capacity (d) and mitochondrial membrane potential (e) was analyzed. The values are the means ± S.E.M from three independent experiments. *P < 0.05 and **P < 0.01 as calculated by one-way ANOVA.

To explore the impact of disrupted mitochondrial fusion on mitochondrial function and evaluate the contribution of key molecules to retinal aging, we knocked down Drp1, Mfn1, and Mfn2 by siRNA, respectively, in normal ARPE-19 cells (Figure 3(a)). Cellular ATP content was decreased after Mfn2 knockdown, while it was not affected after Drp1 and Mfn1 knockdown (Figure 3(b)). Consistently, Mfn2 knockdown increased cellular ROS level, while neither Drp1 nor Mfn1 knockdown had any effect (Figure 3(c)). Meanwhile, we found that Mfn2 knockdown could decrease mitochondrial oxygen consumption (Figure 3(d)) and mitochondrial membrane potential (Figure 3(e)) which may contribute to lower ATP content and increased ROS production.

### 3.4. Alteration of MAPK Signaling Pathway during Retinal Aging

**Figure 4:**
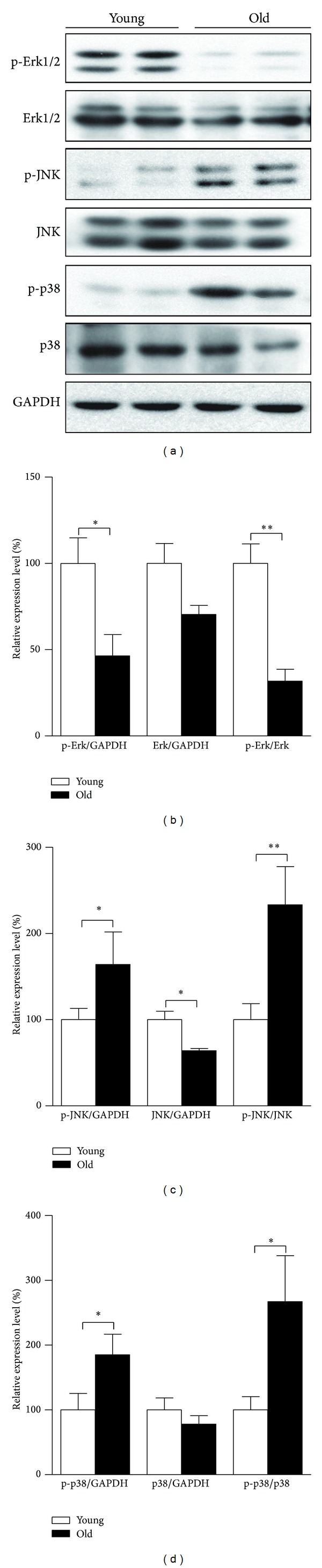
Alterations of MAPK pathway in retinas of young and old rats. SD rats aged five months (young) and 25 months (old) were sacrificed; total proteins of retina tissues were prepared for analysis. MAPK pathway activation statuses were analyzed by Western blot ((a) Western blot image, (b) statistical analysis of p-Erk1/2, (c) statistical analysis of p-JNK, and (d) statistical analysis of p-p38). The values are the means ± S.E.M from 8 rats per group. *P < 0.05 and **P < 0.01 as calculated by Student's *t*-test.

To help further validate that retinal aging is associated with oxidative stress, activation state of classical mitogen-activated protein kinases (MAPKs) including extracellular signal-regulated kinase 1/2 (Erk1/2), c-Jun N-terminal kinase (JNK), and p38 was examined. As shown in Figure 4, considerably increased phospho-JNK (Figures 4(a) and 4(c)) and phospho-p38 (Figures 4(a) and 4(d)) were detected in aged retina tissue, reflecting increased stress stimuli in aged retina. This helps to corroborate our speculation that oxidative stress and raised ROS level occur in aged retina. In addition, both phospho-Erk1/2 and Erk1/2 themselves dropped dramatically in aged retinas (Figures 4(a) and 4(b)), which is in agreement with the expectation of the decline of growth factor signaling with aging.

### 3.5. Elevated Protein O-GlcNAc Modification with Lower Expression of OGA in Aged Retina

**Figure 5:**
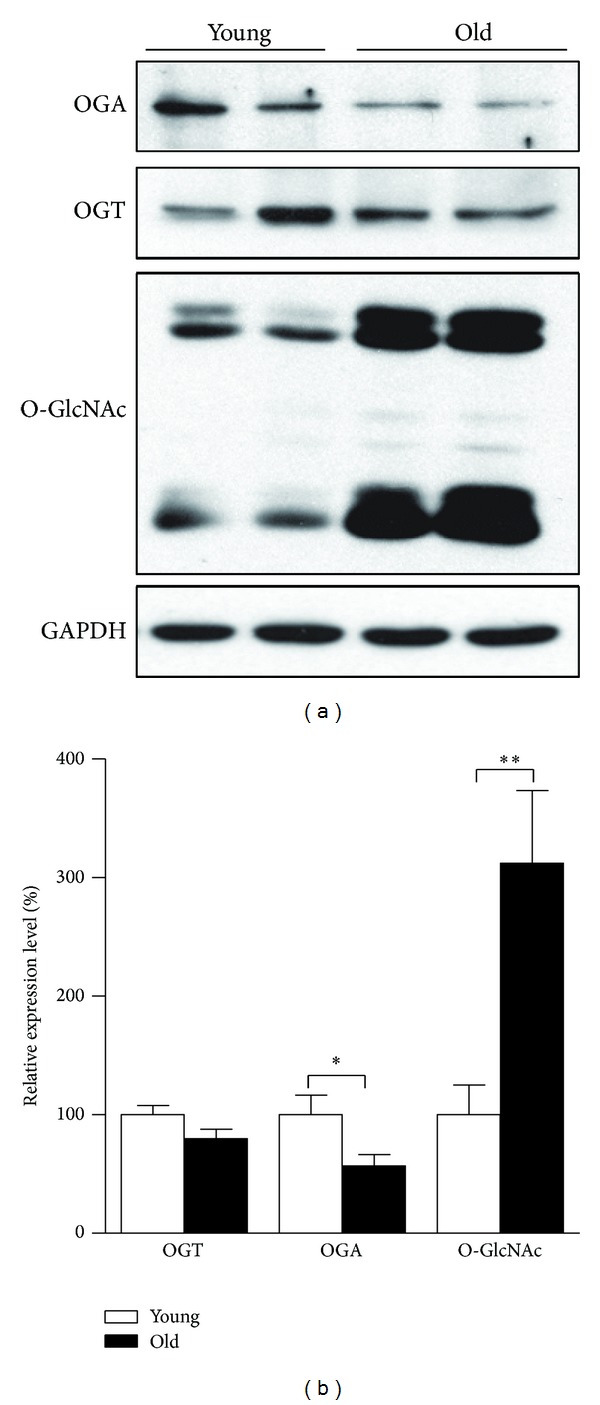
Protein O-GlcNAc modification levels and expression of O-GlcNAc cycling enzymes in retinas of young and old rats. SD rats aged five months (young) and 25 months (old) were sacrificed; total proteins of retina tissues were prepared for analysis. OGA, OGT, and O-GlcNAcylation of proteins were analyzed by Western blot ((a) Western blot image and (b) statistical analysis). The values are the means ± S.E.M from 8 rats per group. *P < 0.05 and **P < 0.01 as calculated by Student's *t*-test.

Cumulative evidence has linked O-GlcNAc modification to stress response, aging, and age-related diseases. To examine how O-GlcNAcylation level is altered in retinal aging, protein expression levels of O-GlcNAc transferase (OGT) and O-GlcNAcase (OGA), as well as the global protein O-GlcNAcylation level in both aged and young retinas was analyzed. As displayed in Figure 5, OGA expression is much lower in aged retina while OGT remains at similar levels between the two groups (Figures 5(a) and 5(b)). Then, in aged retina, the unbalanced pair of enzymes is supposed to drive the O-GlcNAc cycling machinery towards high cumulative yield of protein O-GlcNAc modification. As predicted, O-GlcNAcylation was observed to be approximately threefold higher in aged retinas than in young ones (Figures 5(a) and 5(b)).

### 3.6. Boosting Protein O-GlcNAc Modification in ARPE Cells Increases Mitochondrial Oxygen Consumption and Decreases ROS Level

**Figure 6:**
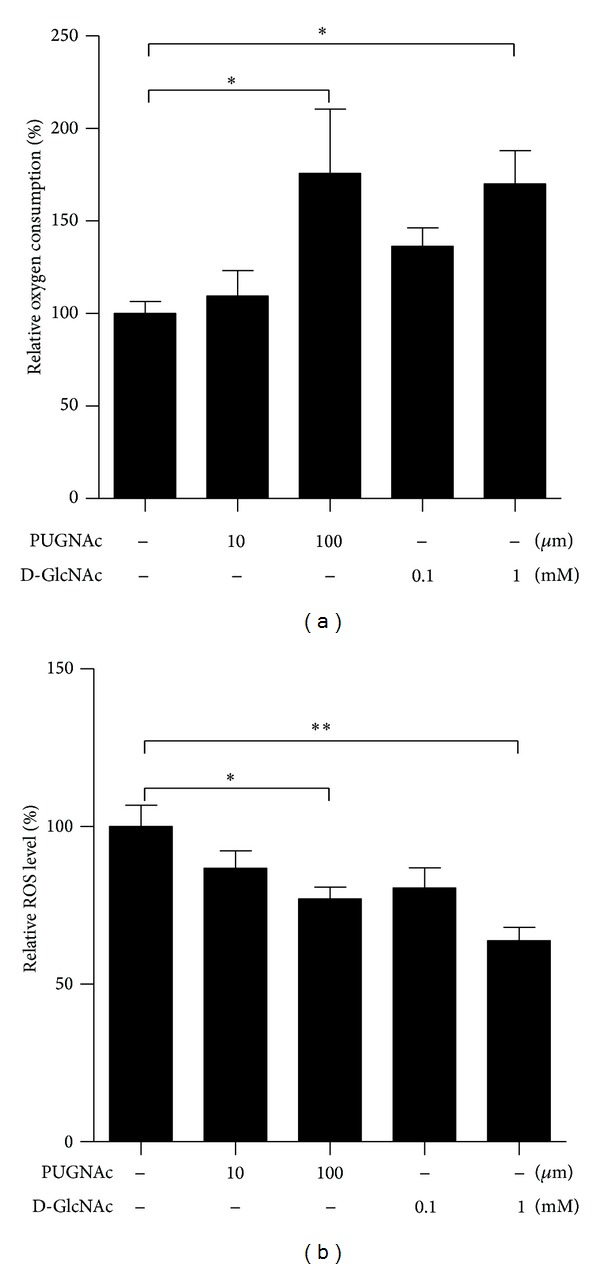
Effects of treatments increasing protein O-GlcNAc modification on mitochondrial function in ARPE-19 cells. ARPE cells were treated with PUGNAc or D-GlcNAc separately for 24 h. Mitochondrial oxygen consumption capacity (a) and ROS content (b) was analyzed. The values are the means ± S.E.M from three independent experiments. *P < 0.05 and **P < 0.01 as calculated by one-way ANOVA.

The above intriguing findings prompted us to wonder about the underlying role of elevated O-GlcNAcylation during retinal aging. To demonstrate the effects of raised O-GlcNAcylation level on normal RPE, we artificially enhanced overall protein O-GlcNAcylation in ARPE-19 cells by treating with PUGNAc, a widely used potent OGA inhibitor, or by directly adding uridine diphosphate N-acetylglucosamine (UDP-GlcNAc), the donor substrate of OGT. Both treatments significantly increased oxygen consumption (Figure 6(a)) and reduced ROS production (Figure 6(b)), though ATP levels were not much affected (data not shown). These results indicate that boosting global protein O-GlcNAcylation level is an effective defense strategy against oxidative stress by reducing ROS level in ARPE cells. It might hence be deduced that elevation of O-GlcNAcylation which occurred in aged retina could also accelerate mitochondrial oxygen consumption and reduce ROS production and may be one of the manifestations of the endeavor trying to slow down retinal aging, or it could also be viewed as an aging-associated adaptation of the retina.

## 4. Discussion

Oxidative stress is viewed as the major factor in retinal aging and age-related retinal degeneration [2, 12, 13, 22, 23]. Most of the current therapeutic strategies for AMD are focused on increasing antioxidant levels to alleviate oxidative damage, and so far limited success has been achieved [12]. Cell regeneration or replacement approach sounds promising yet still under development in the lab [12]. In the present study, we quested for the mechanism of normal retinal aging from the perspective of alterations in mitochondria and global O-GlcNAc modification by comparing retinas from 5-month-old and 25-month-old rats and conducting supportive tests on ARPE-19 cellular models. Our results regarding Mfn2 downregulation causally related to functional decline of mitochondria in retinal aging are consistent with the consensus that abnormal mitochondrial dynamics is an important factor in aging process and aging-associated pathologies converged by a wave of recent findings. Proteins participating in mitochondrial fusion and fission are also involved in a broad range of cellular processes, like mitochondrial metabolism, redox signaling, maintenance of mtDNA integrity, and cell death [24]. Kowald and Kirkwood recently proposed a hypothesis from an evolutionary perspective that mitochondrial fusion is causally involved in the accumulation of mitochondrial mutants during animal aging [25]. Disruption of mitochondrial dynamics not only affects mitochondrial structure but also intrinsically causes mitochondrial dysfunction and contributes to oxidative stress or even cell death as a vital part of the quality-maintenance mechanism [24, 26]. Among all the essential components of mitochondrial fusion/fission apparatus, Mfn2 is found also as critical participant of various cellular processes and undergoes tight regulatory control. Recently, it was shown that JNK phosphorylation of Mfn2 in response to cellular stress leads to ubiquitination and proteasomal degradation of Mfn2, mediating mitochondrial fragmentation and stress-induced apoptosis [27]. On the other hand, Mfn2 deficiency was shown to be associated with active JNK, endoplasmic reticulum stress, enhanced hydrogen peroxide concentration, altered ROS handling, and susceptibility to insulin resistance in liver and muscle [28]. Therefore, loss of Mfn2 and potential decline of other components of mitochondrial dynamics during retinal aging not only impede proper mitochondrial form but also contribute to the deterioration of metabolic and redox homeostasis and increased stress-induced mutation and apoptosis along with aging.

It is becoming increasingly apparent that extensive mitochondrial fragmentation caused by either retarded mitochondrial fusion or hyperactive fission is a mechanistic instigator of aging and pathogenesis or progression of age-related disease, at least partially via the link of oxidative stress. Therefore, our study suggests that manipulation of mitochondrial fusion component Mfn2 could be a potentially promising therapeutic strategy to forestall retinal aging and aged-associated retinal degeneration; yet further investigations are required to explore the practical approach.

In agreement with our results of implication of decreased mitochondrial biogenesis and disrupted mitochondrial fusion in retinal aging, it has been suggested that mitochondrial biogenesis and dynamics are causatively related to diverse central and peripheral neurodegeneration [29], where the emphasis is on the impact of defective mitochondrial biogenesis resulting from lack of PGC1-*α*, master regulator of mitochondria biogenesis and function [30]. Although our judgment of decreased mitochondrial biogenesis is mainly based on the robustly reduced expression of mtTFA, notably the expression of PGC1-*α* did exhibit a tendency to decrease in aged retinas compared to young ones as shown in Figure 1. Modulating mitochondrial biogenesis by pharmaceutical or molecular means might also be effective in retarding retinal aging and age-related retinal degeneration.

The dynamic and reversible O-GlcNAc modification is orchestrated by the interplay of a pair of O-GlcNAc cycling enzymes, OGT and OGA, catalyzing addition to or removal from the serine and threonine residues of proteins, respectively. The hexosamine signaling pathway has emerged as a versatile cellular regulator modulating numerous cellular signaling cascades influencing growth, metabolism, cellular stress, circadian rhythm, and host-pathogen interactions. Emerging researches suggest that abnormal O-GlcNAcylation is involved in chronic diseases of aging including diabetes, cardiovascular disease, neurodegenerative disorders, and cancer [31–33]. However, there is merely sparse evidence by now with regard to the change of O-GlcNAcylation in normal aging. It's recently reported that the higher O-GlcNAcylation level of older mice in diverse tissues including brain, lung, skin, thymus, testis, and liver was explained by abnormal OGT/OGA balance [34]. As for O-GlcNAcylation in the retina, O-GlcNAcylation has lately been implicated in the pathogenesis of diabetic retinopathy (DR) [35–37]. In particular, O-GlcNAc modification of specificity protein 1, a transcriptional factor participating in angiogenesis, is reported to be involved in transcriptional activation of endothelial growth factor A in the pathogenesis of DR [35]. As shown in Figure 5, we found more intense pan-O-GlcNAcylation accompanied by diminished OGA expression in aged retina. In addition, we demonstrated that both chemical inhibition of OGA activity and excessive supply of O-GlcNAcylation donor substrates could promote oxygen consumption and suppress ROS level in ARPE-19 cells.

The relationship between O-GlcNAcylation and mitochondria has been an elusive subject. It is suggested that OGT and O-GlcNAc might be mediating the link between mitochondrial motility and availability of mitochondrial substrates in neurons [38]. Besides, PGC1-*α* was shown to be O-GlcNAcylated [39]; however, the physiological role is not very clear yet. Although the existence of a shorter isoform of OGT encoding a mitochondrially sequestered enzyme with an N-terminal mitochondrion-targeting sequence in mammals besides the long isoform nucleocytoplasmic OGT was reported a decade ago [40], the role that O-GlcNAc modification of mitochondrial proteins plays is still largely unknown. In cardiac myocytes, members of ETC complexes were shown to be O-GlcNAcylated and exposure to high glucose increased these O-GlcNAcylations, leading to impaired mitochondrial function involving activity of respiratory complexes, calcium and ATP [41]. It is also suggested that O-GlcNAcylation of mitochondrial proteins may have a role in normal functioning of mitochondria, and high glucose induced changes in O-GlcNAcylation of mitochondrial proteins may also be associated with mitochondrial dysfunction and insulin resistance in myoblast cells [42]. In neonatal cardiac myocytes, decreased OPA1 protein level and increased O-GlcNAcylation of OPA1 protein by high glucose could lead to mitochondrial dysfunction by increasing mitochondrial fragmentation, decreasing mitochondrial membrane potential, and attenuating the activity of ECT Complex IV [43].

Despite those reports involving O-GlcNAcylation with impaired mitochondrial function, in fact, in cardiovascular system, elevation of O-GlcNAcylation has been viewed as an autoprotective alarm or stress response, and promoting O-GlcNAcylation actually safeguards cell survival under the strike of acute stresses [44]. Augmented O-GlcNAc signaling has been shown to attenuate hypoxic or oxidative stress-induced calcium overload and to mitigate hydrogen peroxide-induced mitochondrial permeability transition pore formation in cardiomyocytes [45]. Glucosamine was reported to protect neonatal rat ventricular myocytes against ischemia/reperfusion injury via increased formation of O-GlcNAc and the protective effects are in part due to enhanced mitochondrial Bcl-2 translocation [46]. Previous study also suggested that O-GlcNAc may enhance cell survival against a diverse array of cellular stresses through regulating DNA damage signaling and repair [47]. Thus, we conjecture that the retina enhances its overall protein O-GlcNAcylation level by adjusting expression of O-GlcNAc cycling enzymes to battle against mitochondrial degeneration and oxidative stress during retinal aging. Notably, we observed a simultaneous elevation of O-GlcNAcylation and activation of p38 and JNK MAP kinases in aged rat retina. It was recently proposed that interaction between OGT and MAPK, mTOR, AMPK, or insulin-AKT signaling cascades at many points provides a nutrient-sensitive balance between growth control and the cellular stress response in feast or famine [48]. Thus, it might be suggested that corecruitment of O-GlcNAc cycling enzymes and MAP kinases together drives the switch impacting pathways of anabolic and catabolic pathways to counteract the increased stress level along with aging.

RPE cells are protected from oxidative stress by a highly elaborate system. Besides the well-studied classical nuclear factor erythroid-2-related factor 2- (Nrf2-) activated antioxidant response consisting of enzymatic and small molecular antioxidants [49], our results imply that O-GlcNAc modification might be putatively bestowed a function of mediating antioxidation or considered as a protective stress-response pathway against ROS in the retinal aging fundus; yet more evidences are needed to support it. Moreover, identifying the profile or the key targets of O-GlcNAc modification during aging seems a vital future research aim to gain insights to considerably add to our understanding of the mystery of aging and the role of O-GlcNAc modification during aging.

## 5. Conclusions

In summary, the current study demonstrates the possible relationship between alterations in mitochondrial homeostasis especially its dynamics and O-GlcNAc modification during retinal aging. Reduced mitochondrial biogenesis and fusion might exacerbate oxidative stress; elevated overall O-GlcNAcylation level might exert protective effects on mitochondrial respiration and redox homeostasis during retinal aging. Accordingly, rescue of Mfn2 loss and modulation of O-GlcNAcylation might represent potential novel therapeutic avenues for the treatment of aging-related retinal diseases. Nevertheless, further studies are called to support these preliminary findings and to facilitate the final translation into effective strategies.
